# Reducing the quality risk of elderly care services in government procurement from market-oriented private providers through *ex ante* policy design: lessons from the principal-agent theory analysis

**DOI:** 10.1186/s12913-020-05994-w

**Published:** 2020-12-10

**Authors:** Huan Song, Sihang Yu, Tao Sun

**Affiliations:** Zhou Enlai School of Government, Nankai University, No.38 Tongyan Road, Jinnan Campus, Tianjin, 300350 China

**Keywords:** Quality of elderly care services, Government procurement, Market-oriented private providers, *Ex ante* policy design, Principal-agent theory

## Abstract

**Background:**

Government procurement of elderly care services from market-oriented private providers has become an important way to respond to the growing demands of elderly care. However, the government cannot accurately identify the actual quality efforts of these providers, and the government pursues social benefits while the providers pursue economic interests. The existence of asymmetric information and goal divergence increases the quality risk of services. From the perspective of maximizing the government’s net benefits, this study aimed to analyze how to reduce the quality risk through *ex ante* policy design.

**Methods:**

On the basis of the principal-agent theory, this study defined the asymmetric information of market-oriented private providers’ efforts on quality as a random variable, and constructed the theoretical model in the case of asymmetric information to compare with the one in the reference case of complete information, in both of which the government is the principal and market-oriented private providers are the agents. And the models also introduced several parameters to describe key factors that affect the contract results, including the physical health of the elderly, the spillover benefits to the government and market-oriented private providers, and the market risks.

**Results:**

The optimal results of the models in the two cases were obtained respectively, and the validity of the theoretical models was verified in a numerical example. Taking the case of complete information as the basic frame of reference, the difference of the optimal results in both cases showed the extent of negative impacts of asymmetric information, and highlighted the role of *ex ante* policy design in minimizing asymmetric information and reducing its negative impacts. Some *ex ante* policies that can improve the supervision of market-oriented private providers and their quality efforts, as well as have positive effects on key factors, were also recommended.

**Conclusions:**

The government should attach importance to *ex ante* policy design to reduce the quality risk of elderly care services supplied by market-oriented private providers in government procurement. Our study provides main framework and critical directions for *ex ante* policy design, which is conducive to the realization of real and sustained quality improvement.

## Background

With the rapid aging of the population, many governments around the world are under pressure to establish a care service system for the elderly. It is reported that the number of elderly people is expected to rise from 524 million in 2010 to 1.5 billion by 2050 globally, compounded by a growing dependency ratio due to declining fertility rates and increased longevity of the elderly [1]. For an individual, the likelihood of needing care by the age of 65 exceeds 35% and increases exponentially from the age of 80 [2, 3]. However, because of socioeconomic changes, the role of the family in shouldering the responsibilities and burdens of caring for the elderly is eroding [4]. In the face of escalating demands of elderly care and the resulting financial pressure, relying solely on the supply of the public sector has also shown its inevitable fragility and finiteness [5]. To cope with the challenges posed by the demand side, under the market mechanism, government procurement of elderly care services from market-oriented private providers has gradually become a common approach adopted by many states on the supply side [6, 7].

Advocates typically argue that the involvement of market-oriented private providers in government procurement allows for greater flexibility in adapting to the diversity and variability of demand than the public sector alone, and they often have greater motivation to shift resources to more cost-effective types of care [8, 9]. Critics, on the other hand, stress that there is a substantial risk that market-oriented private providers will prioritize economic interests over the quality of care services, so that maintaining a desired quality level after the government signs a purchase contract with them may be a concern [10, 11]. Several studies have indicated that quality can indeed be sacrificed in the pursuit of cost reduction, especially if the providers of elderly care services are for-profit [12, 13], and government departments lack comprehensive quality monitoring capabilities, resulting in imperfect supervisory measures [14, 15]. To better meet the demands of elderly care and achieve the government’s procurement goals, enough attention should be paid to policy-level design to reduce the quality risk of elderly care services supplied by market-oriented private providers.

Existing researches on the quality of elderly care services mainly focused on the *ex post* control, and the results are reflected in the measurement of objective quality indicators [16–18]. One of the common indicators is the mortality rate. As a non-contractible dimension, it is usually used to reflect quality, indicating measures to reduce the risk of quality degradation when the provision of care services is marketized [13]. In a review of 80 studies, a meta-analysis also presented some objective indicators such as physical health, basic environment, and technical conditions to reflect the possible quality problems in the provision of elderly care services by market-oriented private providers [19]. However, some scholars believe that the quality of care services is multidimensional and often subjective, and it is difficult to implement and define a set of effective and representative indicators to reflect the final quality results [10, 20]. Moreover, studies have shown that if the quality is not verifiable, the moral hazard of *ex post* quality degradation is inevitable [21]. Therefore, it is necessary to put greater weight on *ex ante* policy to control the service quality.

Regarding the quality control of elderly care services, *ex ante* policy design usually takes the form of standardizing administrative procedures, such as limiting the scope of activities and defining obligations to be followed [22, 23]. However, privatization often leads to a decline in transparency and a loss of democratic accountability [24, 25]. After the government signs a purchase contract with market-oriented private providers, the government usually cannot accurately observe their actual efforts on quality that are kind of personal information and it’s easy to be concealed, so the issue of moral hazard often occurs. Compared with the external monitoring system, the moral hazard caused by the private pursuit of self-interest in the public-private partnership is more difficult to solve through the public control system [26, 27]. The existence of asymmetric information and goal divergence makes the government’s *ex ante* policy design need to be more comprehensive, proactive and pragmatic to deal with the service quality issues of market-oriented private providers.

The purposes of government procurement are to meet the enormous demands of elderly care services under the tight budget constraint, and to achieve the expansion of service coverage and the improvement of service quality from a procurement contract linked to private ownership and marketization. However, the quality risk after signing the contract caused by asymmetric information and goal divergence is bound to affect the ultimate realization of the contract objectives. The difference of the optimal results in the cases of asymmetric information and complete information can clearly reflect the specific extent of such influence to highlight the disadvantage of asymmetric information. In this regard, few scholars have compared the results of the two cases to seek a breakthrough in *ex ante* policy design. From the perspective of maximizing the government’s net benefits, this study aimed to fill this research gap to emphasize the role of *ex ante* policy design and explore some promising and strong measures to minimize asymmetric information and reduce its negative impacts.

The principal-agent theory is a widely used method to model the process of optimization design of government contracts in the presence of moral hazard [28]. It has been applied not only to the study of fair competition [29] and legal rules [30] in government procurement contracts but also to the design of government employment contracts [31] and tax incentive contracts [32]. On the basis of the principal-agent theory, our study defined the asymmetric information of market-oriented private providers’ efforts on quality as a random variable, and constructed the theoretical models in the case of asymmetric information and the reference case of complete information, in both of which the government is the principle and market-oriented private providers are the agents. Then, the optimal results in both cases, the validity of the theoretical models, and related findings were presented. Finally, critical and effective measures were discussed and recommended for *ex ante* policy design, which aimed to provide worthwhile lessons for policymakers.

## Methods

### Model theoretical basis

The core task of principal-agent theory is to design an optimal contract to motivate the agents based on the principal’s interests [33]. Since both the principal and the agents are rational economic subjects whose behavior goals are to maximize their own interests, which makes the problem of moral hazard often occur in the case of asymmetric information [34]. It can be seen that asymmetric information and goal divergence are two key issues in the principal-agent relationship [35, 36]. First, asymmetric information makes it impossible for the principal to directly observe the specific action choice of the agents, which leads to the possibility of the agents’ opportunistic practices of deceit and fraud [37]. Second, goal divergence between the principal and the agents is inevitable, because in essence, the interests of the principal depend on the costs (efforts) of the agents, while the interests of the agents depend on the costs (payments) of the principal [38]. As a consequence, the agents can make use of asymmetric information to reduce efforts and pursue the maximization of their own interests while ignoring or damaging the interests of the principal [33].

Some studies have pointed out that asymmetric information can lead to an increase in transaction costs, so that a bounded rational principal may choose to satisfy the status quo by weighing the marginal cost against the marginal benefit of monitoring strategies [39, 40]. Therefore, to motivate the agents to choose the behavior consistent with the desire of the principal, the design of the incentive mechanism is extremely important in principal-agent relationships [40]. The principal-agent model focuses on the responsive decision of the agents to the principal’s goal and on how to design an optimal contract from the perspective of the principal’s interests [33, 37]. Generally speaking, in the principal-agent model, the optimal contract should satisfy two conditions: one is incentive compatibility constraint, in which the interests obtained by the agents from the behaviors expected by the principal are not less than those obtained from any other behaviors; the other is participation constraint, in which the interests obtained by the agents from accepting the contract are not less than those obtained from rejecting the contract, that is, the reservation profit [41, 42].

Since the 1990s, the concept of principal-agent has been introduced into the research of policymaking, most notably mentioned is that the role of various funding agencies responsible for implementing policy research can be a double-edged sword for policymakers [43]. With the development of science and technology, researchers have tried to integrate the principal-agent theory with data-driven decision-making to provide more precise policy options for future policy formulation [44]. And now, it has been increasingly applied to emerging policy issues, such as analyzing how to quickly mobilize enterprises to join in low-carbon activities by providing subsidies [45], combining the fairness preference theory to calculate the optimal incentives of the government in public-private partnership projects [46], and deriving an agent-based model for policymaking on energy-efficiency retrofit in the building sector under its theoretical framework [47].

In the area of government procurement, predecessors have used this theory to study various agency problems, including assignment of responsibilities [23], negotiation of purchase price [24], and evaluation of the effectiveness of procurement strategies [48]. In this study, we made further efforts to apply the principal-agent theory to analyze the issue of quality risk of elderly care services in government procurement from market-oriented private providers. Furthermore, we constructed a reference case of complete information to illustrate the extent of negative impacts of asymmetric information on the optimal results. Unlike the optimal contract in the case of asymmetric information, which needs to meet the above two constraints, the optimal contract in the case of complete information only needs to meet the participation constraint because of the information advantage [41].

### Model notations and assumptions

Market-oriented private providers’ efforts in the quality of elderly care services are the personal information that cannot be accurately observed by the government. It’s supposed to be represented by a random variable *x*. Under the effort level *x*, the price *p*(*x*) and quantity *q*(*x*) of services purchased can be determined by the government and market-oriented private providers on both sides of the contract. Thus, *p*(*x*) ⋅ *q*(*x*) is the total cost of the government and a part of the income of market-oriented private providers. Table 1 shows the full picture of the benefits and costs of both parties.

**Table 1:** Benefits and costs matrix for stakeholders

Stakeholders	Benefits	Costs
Government	*U*(*λ*, *q*(*x*))	*p*(*x*)*q*(*x*)
Market-oriented private providers	*p*(*x*)*q*(*x*) + *w*(*σ*, *ε*, *x*)	*C*(*θ*, *q*(*x*), *x*)

For the government, *U*(*λ*, *q*(*x*)) reflects social benefits. On the one hand, the quantity of services purchased directly determines whether the demands of the elderly are satisfied, including the accessibility and fairness of services. On the other, the signing of the contract has positive spillover effects on the society, including achieving social justice, maintaining family harmony, expanding service markets, increasing employment channels and promoting technological innovation [7, 49, 50], which are also parts of the government’s benefits, represented by the parameter *λ*(*λ* > 1).

For market-oriented private providers, the other part of the income is *w*(*σ*, *ε*, *x*) derived from non-price factors. The parameter *σ*(*σ* > 1) describes the spillover benefits of caring for the elderly, including corporate reputation, social recognition, government and public dependence [51, 52]. And the parameter *ε*(*ε* > 0) explains the market risks from finance and competitors that inevitably have impacts on the actual returns of market-oriented private providers [53]. The total cost is expressed as *C*(*θ*, *q*(*x*), *x*), which is influenced by the effort level and the quantity of care services supplied. The parameter *θ*(*θ* > 1) is introduced to represent the physical health of the elderly, because the costs of providing care for self-care, semi-disabled and disabled elderly are different [5].

Accordingly, the government’s expected net benefits *S* is obtained as

1 $$ S\left(p(x),q(x)\right)=E\left[U\left(\lambda, q(x)\right)-p(x)q(x)\right], $$

and market-oriented private providers’ profit *π* is derived as

2 $$ \pi \left(p(x),q(x),x\right)=p(x)q(x)-C\left(\theta, q(x),x\right)+w\left(\sigma, \varepsilon, x\right). $$

To achieve a fit between the model and real issue, and to ensure the existence of the optimal solution, before establishing the principal-agent model, we proposed the following assumptions about the functional properties of some variables by referring to previous studies [54, 55].

1. The effort level *x* is a random variable with a support [0, *b*],where 0 ≤ *b* ≤ 1.
2. The distribution function of *x* is *Y*(*x*), and *y*(*x*) is the derivation of *Y*(*x*), where they satisfy $\frac{d}{dx}\left(\frac{1-Y(x)}{y(x)}\right)<0$.
3. The revenue function of the government *U*(*λ*, *q*(*x*)) is an increasing and concave function, that is, $\frac{dU\left(\lambda, q(x)\right)}{dq}>0,\frac{d^2U\left(\lambda, q(x)\right)}{dq^2}<0$.
4. The non-price income function of market-oriented private providers *w*(*σ*, *ε*, *x*) is increasing with respect to *x*.
5. The costs of market-oriented private providers *C*(*θ*, *q*(*x*), *x*) should satisfy $\frac{\partial C\left(\theta, q(x),x\right)}{\partial x}<0$, $\frac{\partial^2C\left(\theta, q(x),x\right)}{\partial x\partial q}<0$, $\frac{\partial^2C\left(\theta, q(x),x\right)}{\partial {q}^2}\ge 0$, $\frac{\partial^3C\left(\theta, q(x),x\right)}{\partial x\partial {q}^2}\le 0$, $\frac{\partial^3C\left(\theta, q(x),x\right)}{\partial {x}^2\partial q}\ge 0$.

### Model construction

The model is constructed to maximize the government’s expected net benefits and ensure market-oriented private providers’ profit. To highlight the influence of asymmetric information, the reference case of complete information is also presented. The models in the two cases are formulated as follows.

**Case 1** Model under asymmetric information.

The government cannot accurately assess the actual quality efforts of market-oriented private providers. Since market-oriented private providers are profit-driven, they may express the effort level *y* to the government to get higher purchase price and quantity, where *x* < *y*. To motivate market-oriented private providers to tell the truth, the incentive compatibility constraint should be designed as

3 $$ \pi \left(p(x),q(x),x\right)\ge \pi \left(p(y),q(y),x\right),\forall x,y\in \left[0,b\right]. $$

Market-oriented private providers often have other investment options in the market. They are encouraged and provide elderly care services only if the reservation profit *π*_0_ (the profit of another alternative investment project) could be obtained (at least). So, the participation constraint should be given as

4 $$ p(x)q(x)-C\left(\theta, q(x),x\right)+w\left(\sigma, \varepsilon, x\right)\ge {\pi}_0,\forall x\in \left[0,b\right]. $$

Therefore, the principal-agent model in Case 1 can be formulated as

5 $$ \left\{\begin{array}{l}\underset{\left(p\left(\cdot \right),q\left(\cdot \right)\right)}{\mathit{\max}}E\left[U\left(\lambda, q(x)\right)-p(x)q(x)\right]\\ {}s.t.\\ {}p(x)q(x)-C\left(\theta, q(x),x\right)+w\left(\sigma, \varepsilon, x\right)\ge p(y)q(y)-C\left(\theta, q(y),x\right)+w\left(\sigma, \varepsilon, y\right),\\ {}\forall x,y\in \left[0,b\right]\\ {}p(x)q(x)-C\left(\theta, q(x),x\right)+w\left(\sigma, \varepsilon, x\right)\ge {\pi}_0,\forall x\in \left[0,b\right]\end{array}\right.. $$

**Case 2** Model under complete information.

In this case, market-oriented private providers’ efforts on quality are clearly known to the government. The government’s procurement contract for elderly care services only needs to meet market-oriented private providers’ reservation profit.

Therefore, the principal-agent model in Case 2 can be formulated as

6 $$ \left\{\begin{array}{l}\underset{\left(p\left(\cdot \right),q\left(\cdot \right)\right)}{\mathit{\max}}E\left[U\left(\lambda, q(x)\right)-p(x)q(x)\right]\\ {}s.t.\\ {}p(x)q(x)-C\left(\theta, q(x),x\right)+w\left(\sigma, \varepsilon, x\right)\ge {\pi}_0,\forall x\in \left[0,b\right]\end{array}\right.. $$

## Results

### Model solution

To get the optimal solution of the model, propositions are presented together with findings following them in this subsection. The process of proof draws on the experience of some earlier studies, such as those published by Chen and Hong [54], Mu, Lan and Tang [55].

**Case 1** Model under asymmetric information.

**Proposition 1** The incentive compatibility constraint (3) can be written as

7 $$ \left\{\begin{array}{l}\frac{dp(x)}{dx}\cdot q(x)+\frac{dq(x)}{dx}\cdot p(x)+\frac{dw\left(\sigma, \varepsilon, x\right)}{dx}=\frac{\partial C\left(\theta, q(x),x\right)}{\partial q}\cdot \frac{dq(x)}{dx}\\ {}\frac{dq(x)}{dx}>0\end{array}\right., $$

and the participation constraint (4) can be expressed as

8 $$ p(0)q(0)-C\left(\theta, q(0),0\right)+w\left(\sigma, \varepsilon, 0\right)={\pi}_0. $$

Therefore, Model (5) is equivalent to

9 $$ \left\{\begin{array}{l}\underset{\left(p\left(\cdot \right),q\left(\cdot \right)\right)}{\mathit{\max}}E\left[U\left(\lambda, q(x)\right)-p(x)q(x)\right]\\ {}s.t.\\ {}\frac{dp(x)}{dx}\cdot q(x)+\frac{dq(x)}{dx}\cdot p(x)+\frac{dw\left(\sigma, \varepsilon, x\right)}{dx}=\frac{\partial C\left(\theta, q(x),x\right)}{\partial q}\cdot \frac{dq(x)}{dx},\forall x\in \left[0,b\right]\\ {}\frac{dq(x)}{dx}>0,\forall x\in \left[0,b\right]\\ {}p(0)q(0)-C\left(\theta, q(0),0\right)+w\left(\sigma, \varepsilon, 0\right)={\pi}_0\end{array}\right.. $$

**Proposition 2** If (*p*^∗^(⋅), *q*^∗^(⋅)) is the optimal solution of Model (5), then there exists

10 $$ \frac{dU\left(\lambda, {q}^{\ast }(x)\right)}{dq^{\ast }}=\frac{\partial C\left(\theta, {q}^{\ast }(x),x\right)}{\partial {q}^{\ast }}-\frac{1-Y(x)}{y(x)}\cdot \frac{\partial^2C\left(\theta, {q}^{\ast }(x),x\right)}{\partial x\partial {q}^{\ast }} $$

and

11 $$ {p}^{\ast }(x)=\frac{C\left(\theta, {q}^{\ast }(x),x\right)-w\left(\sigma, \varepsilon, x\right)-{\int}_0^x\frac{\partial C\left(\theta, {q}^{\ast }(t),t\right)}{\partial t} dt+{\pi}_0}{q^{\ast }(x)}. $$

#### Findings

1. The transformed incentive compatibility constraint (7) in Proposition 1 states that the marginal benefit must be equal to the marginal cost to ensure the existence of the optimal solution, and if market-oriented private providers increase their efforts on quality, the quantity of elderly care services purchased by the government will expand, that is, as the quality of services improves, the care demands of more elderly people will be satisfied.
2. Moreover, in Proposition 1, the participation constraint changes from inequality (4) to equality (8), indicating that the government, as a rational economic subject, chooses the minimum price to satisfy market-oriented private providers’ participation threshold to maximize its own net benefits.
3. Based on the equalities (10)–(11) in Proposition 2, the optimal solution of Model (5) can be obtained. And accordingly, we could infer that in addition to the effort level *x*, the parameter *λ*, *σ*, *ε* and *θ* are all related to *p*^∗^(⋅) and *q*^∗^(⋅). The following part of model validation will discuss their specific effects on the optimal solution.

**Case 2** Model under complete information.

**Proposition 3** Model (6) is equivalent to

12 $$ \left\{\begin{array}{l}\underset{\left(p\left(\cdot \right),q\left(\cdot \right)\right)}{\mathit{\max}}E\left[U\left(\lambda, q(x)\right)-p(x)q(x)\right]\\ {}s.t.\\ {}p(x)q(x)-C\left(\theta, q(x),x\right)+w\left(\sigma, \varepsilon, x\right)={\pi}_0,\forall x\in \left[0,b\right]\end{array}\right.. $$

**Proposition 4** If (*p*^∗∗^(⋅), *q*^∗∗^(⋅)) is the optimal solution of Model (6), then there exists

13 $$ \frac{dU\left(\lambda, {q}^{\ast \ast }(x)\right)}{dq^{\ast \ast }}=\frac{\partial C\left(\theta, {q}^{\ast \ast }(x),x\right)}{\partial {q}^{\ast \ast }} $$

and

14 $$ {p}^{\ast \ast }(x)=\frac{C\left(\theta, {q}^{\ast \ast }(x),x\right)-w\left(\sigma, \varepsilon, x\right)+{\pi}_0}{q^{\ast \ast }(x)}. $$

#### Findings

1. In this case, the quality efforts of market-oriented private providers are complete information for the government, so that the government could further coordinate purchase price with market-oriented private providers if the participation constraint is an inequation. Due to the government’s goal of maximizing net benefits, the participation constraint eventually equals to market-oriented private providers’ reservation profit, as presented in Proposition 3.
2. By comparing equality (10) and equality (13), we found that the marginal benefit for the government in Case 1 is smaller than in Case 2. $-\frac{1-Y(x)}{y(x)}\cdot \frac{\partial^2C\left(\theta, {q}^{\ast }(x),x\right)}{\partial x\partial {q}^{\ast }}$ can be seen as an information cost for the government to encourage market-oriented private providers to “tell the truth”.
3. The obvious difference between *p*^∗^(⋅) and *p*^∗∗^(⋅) in Proposition 2 and Proposition 4 is $-{\int}_0^x\frac{\partial C\left(\theta, {q}^{\ast }(t),t\right)}{\partial t} dt$, which indicates that instead of enjoying a higher price, market-oriented private providers will pay for “lying”. The specific impacts of asymmetric information on the contract price will be further discussed in the following part of model validation.

### Model validation

In this section, a numerical example is provided to verify the validity of the theoretical models. Following the model assumptions mentioned above, the specific function expressions of the variables are shown in Table 2, which are simple and without loss of generality. Besides, we used MATLAB software to visualize the simulation results and pointed out the corresponding findings.

**Table 2:** Function expressions of variables

Expression and definition	Predicated model assumption
*x* follows a linear uncertainty distribution	√Assumption (1)
$$ y(x)=\left\{\begin{array}{l}1,0<x<1\\ {}0,\begin{array}{c}\end{array} others\end{array}\right. $$	√Assumption (2)
*U*(*λ*, *q*(*x*)) = *λ* ln *q*(*x*)	√Assumption (3)
*w*(*σ*, *ε*, *x*) = *σe*^−*ε*^*x*^2^	√Assumption (4)
*C*(*θ*, *q*(*x*), *x*) = *θ* ⋅ *q*(*x*)/(*x* + 3)	√Assumption (5)

According to equalities (1)–(2) and Table 2, the government’s expected net benefits *S* can be written as

15 $$ S\left(p(x),q(x)\right)=E\left[\lambda \ln q(x)-p(x)q(x)\right], $$

and market-oriented private providers’ profit *π* can be expressed as

16 $$ \pi \left(p(x),q(x),x\right)=p(x)q(x)-\theta \cdot \frac{q(x)}{x+3}+\sigma {e}^{-\varepsilon }{x}^2. $$

At this point, the models and optimal solutions in both cases are presented as follows.

**Case 1** Model under asymmetric information.

The principal-agent model in Case 1 can be formulated as

17 $$ \left\{\begin{array}{l}\underset{\left(p\left(\cdot \right),q\left(\cdot \right)\right)}{\mathit{\max}}E\left[\lambda \ln q(x)-p(x)q(x)\right]\\ {}s.t.\\ {}p(x)q(x)-\theta \cdot \frac{q(x)}{x+3}+\sigma {e}^{-\varepsilon }{x}^2\ge p(y)q(y)-\theta \cdot \frac{q(y)}{x+3}+\sigma {e}^{-\varepsilon }{y}^2,\\ {}\forall x,y\in \left(0,1\right]\\ {}p(x)q(x)-\theta \cdot \frac{q(x)}{x+3}+\sigma {e}^{-\varepsilon }{x}^2\ge {\pi}_0,\forall x\in \left(0,1\right]\end{array}\right.. $$

According to Propositions 1-2, the optimal solution can be obtained as

18 $$ \left\{\begin{array}{l}{q}^{\ast }(x)=\frac{\lambda }{4\theta}\cdot {\left(x+3\right)}^2\\ {}{p}^{\ast }(x)=\frac{\theta }{\lambda}\cdot \frac{2\lambda x-4\sigma {e}^{-\varepsilon }{x}^2+3\lambda +4{\pi}_0}{{\left(x+3\right)}^2}\end{array}\right.. $$

**Case 2** Model under complete information.

The principal-agent model in Case 2 can be formulated as

19 $$ \left\{\begin{array}{l}\underset{\left(p\left(\cdot \right),q\left(\cdot \right)\right)}{\mathit{\max}}E\left[\lambda \ln q(x)-p(x)q(x)\right]\\ {}s.t.\\ {}p(x)q(x)-\theta \cdot \frac{q(x)}{x+3}+\sigma {e}^{-\varepsilon }{x}^2={\pi}_0,\forall x\in \left(0,1\right]\end{array}\right.. $$

According to Propositions 3-4, the optimal solution can be obtained as

20 $$ \left\{\begin{array}{l}{q}^{\ast \ast }(x)=\frac{\lambda }{\theta}\cdot \left(x+3\right)\\ {}{p}^{\ast \ast }(x)=\frac{\theta }{\lambda}\cdot \frac{\lambda -\sigma {e}^{-\varepsilon }{x}^2+{\pi}_0}{x+3}\end{array}\right.. $$

**Fig. 1:**
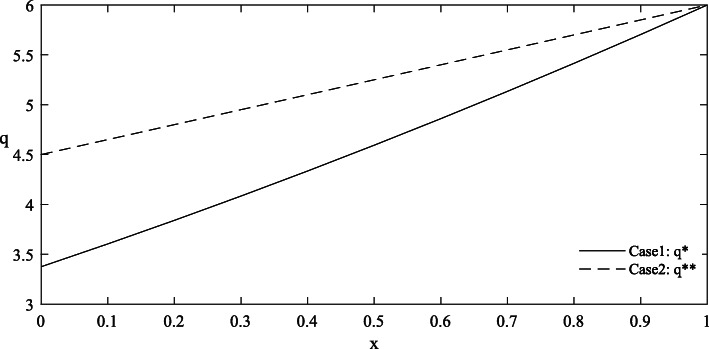
Comparison of functional images of the optimal quantity in Case 1 and Case 2

**Fig. 2:**
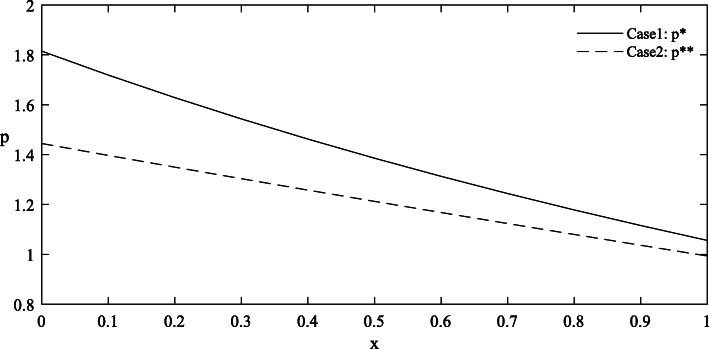
Comparison of functional images of the optimal price in Case 1 and Case 2

**Fig. 3:**
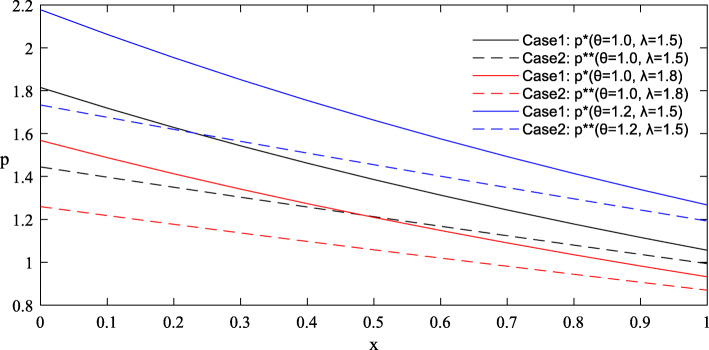
Influence of parameters *θ* and *λ* on the optimal price in Case 1 and Case 2

**Fig. 4:**
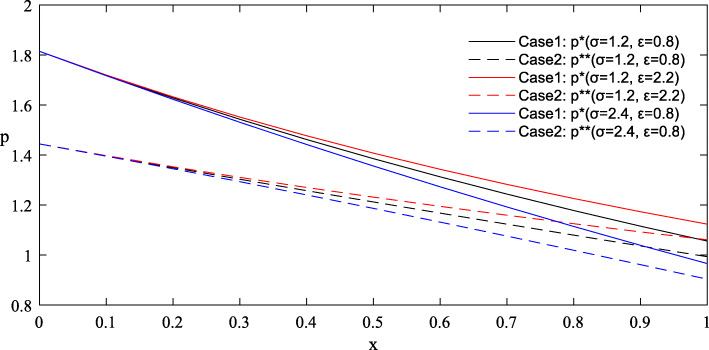
Influence of parameters *σ* and *ε* on the optimal price in Case 1 and Case 2

Figures 1 and 2 provide the simulated function images of the optimal solutions in both cases with taking {*θ* = 1.0, *λ* = 1.5, *σ* = 1.2, *ε* = 0.8, *π*_0_ = 5} for simplicity. Additionally, to highlight the influence of parameters on the optimal price, reference groups {*θ* = 1.2, *λ* = 1.8} and {*σ* = 2.4, *ε* = 2.2} are set in Figs. 3 and 4 respectively.

#### Findings

1. Complete information is more beneficial to the government than asymmetric information. As shown in Figs. 1 and 2, under the condition of complete information, the government can purchase more care services for the elderly at a lower price. But it’s worth noting that the advantage of complete information shrinks as the level of effort increases.
2. The trends of the optimal solutions in Figs. 1 and 2 show that the negative impacts of asymmetric information can be further amplified at a relatively low level of effort, which indicates that improving the quality efforts of market-oriented private providers can help expand the quantity of government procurement and reduce the price cost.
3. The differentiated values of the parameters in Figs. 3 and 4 stress that any improvements in these factors, including the physical health of the elderly, the spillover benefits to the government and market-oriented private providers, and the market risks, are conducive to the reduction of the contract price, so as to realize the government’s cost savings.
4. Given that the optimal quantity in both cases is influenced positively by the parameter *λ* and negatively by the parameter *θ*, the implementation of some measures involving taking advantage of the social spillover benefits of government procurement, grasping the health information of the elderly and striving to improve their physical health, can drive the government to increase the purchase of elderly care services with limited budgets, thereby expanding the coverage of services and meeting the demands of more elderly people.

## Discussion

The results of the principal-agent model in the two cases have shown that complete information is better than asymmetric information for both the government and market-oriented private providers. The realization of complete information means that the government can accurately observe and identify the true level of quality efforts of market-oriented private providers. However, it seems difficult to achieve in practice. The unpredictability and unobservability of quality efforts often make them impossible to be quantified and clearly written into the contract, because even if there is a breach of contract, it cannot be verified, while what the government only knows is usually the final execution results of the contract. Moreover, market-oriented private providers cannot change their nature of pursuing economic interests, and will inevitably cover up or deceive their actual quality efforts.

Compared with the idealized case of complete information, in the case of asymmetric information, the government can still optimize the results of the contract through *ex ante* policy design based on participation constraint and incentive compatibility constraint. The purpose of this study is to maximize the government’s net benefits. On the one hand, the model results in the case of complete information are used as the basic frame of reference to highlight the negative impacts of asymmetric information on the optimal results, and to explain the importance of *ex ante* policy design. On the other, according to several *Findings* derived from the models in the two cases, some important implications for policy design from the perspective of minimizing asymmetric information and reducing its negative impacts are given as follows.

Regarding minimizing asymmetric information, the topics involving supervision and management are inevitable. The principal has to pay a certain cost for the agents to “tell the truth”, and the agents also require to pay a corresponding price for “their lies”. Both parties weigh costs against benefits, which requires the setting of appropriate rules to realize their respective interests. Considering the administrative cost of government supervision, combined with the market environment, it is recommended to perfect the quality evaluation mechanism of the elderly and their families as service users, and adopt the installment payment method in the contract to adjust the purchase price and quantity in real time according to the feedback results [13]. Meanwhile, the government’s procurement process should be finely managed, especially the comprehensive evaluation of the qualifications of market-oriented private providers in advance. Medical and other technical care services for the elderly must be handed over to professionally qualified providers to ensure the quality of services [15].

The negative impacts of asymmetric information on the optimal results are mainly related to the quality effort level of market-oriented private providers and the value of several key factors. First, Figs. 1 and 2 have illustrated that as the effort level increases, the curve position of optimal results in the case of asymmetric information is gradually close to that in the case of complete information, indicating that the negative impacts of asymmetric information are weakened. In other words, when the effort level is relatively high, the degree of change in the optimal results caused by the symmetry of information will be reduced. Therefore, some favorable policies that are conducive to improving the quality effort level of market-oriented private providers should be provided. For example, through subsides, the government can actively promote their cooperation with professional medical institutions to improve nursing skills, and encourage university-industry joint research in the field of elderly care to incubate more high-tech auxiliary nursing devices for the elderly [53].

Second, some positive measures involving key factors, including the establishment of an enabling environment and the acquisition of health information of the elderly, should also be considered. In terms of the enabling environment, it is mainly to increase spillover benefits to the government and market-oriented private providers, and reduce market risks, such as actively playing the role of government procurement in promoting social fairness and justice, cultivating the social responsibility and service brand of market-oriented private providers, and standardizing the investment and financing environment [53, 56, 57]. Concerning the health information of the elderly, combined with the background of the current digital age, it seems more effective to use the Internet cloud platform to establish a personal health database and community-based care needs registration system to achieve targeted health improvement, and take into account the demands of different elderly groups.

The systematic construction of *ex ante* policies derived from the above analysis is bound to reduce the quality risk of elderly care services supplied by market-oriented private providers, which also lays the foundation for further marketization. However, continuous improvements in quality and future marketization require a more complete *ex ante* policy framework to support it. To this end, one could extend our research by analyzing more stakeholders’ interactions with the government to explore more detailed policy implications for *ex ante* control, such as introducing elderly users and their families as the third stakeholder. Besides, because the research topics involve some variables which are difficult to quantify, such as spillover benefits and quality efforts, relevant data cannot be obtained. Our study chose the analysis method of mathematical models. But in general, it provides a paradigm reference and theoretical framework for future research combining empirical data and cases.

## Conclusion

Government procurement of elderly care services from market-oriented private providers takes a greater risk of quality in the context of asymmetric information and goal divergence. The issue of service quality is not only related to the satisfaction of the care demands of the elderly, but also reflects the governance capacity of the government. *Ex ante* policy design plays a vital role in reducing the quality risk of the services supplied by market-oriented private providers. Many proactive and representative measures have been extracted from the construction and solution of the principal-agent model. The results of this study provide important implications on the main directions of *ex ante* policy design in minimizing asymmetric information and reducing its negative impacts, so as to achieve the real and sustained quality improvement of elderly care services.

## Data Availability

Data sharing is not applicable to this article as no datasets were generated or analyzed during the current study. The values of the variables and parameters involved were described in the article.

## Abbreviation

MATLAB: Matrix Laboratory
